# Design and Implementation of a Fully-Actuated Integrated Aerial Platform Based on Geometric Model Predictive Control

**DOI:** 10.3390/mi13111822

**Published:** 2022-10-25

**Authors:** Chuanbeibei Shi, Yushu Yu

**Affiliations:** 1 School of Mechatronical Engineering, Beijing Institute of Technology, Beijing 100081, China; 2 The Edward S. Rogers Sr. Department of Electrical and Computer Engineering, University of Toronto, Toronto, ON M5S 3G4, Canada

**Keywords:** integrated operation of multiple unmanned aerial vehicles, six-dimensional fully actuation, model predictive control, bus communication, real-world flight test

## Abstract

Unlike individual unmanned aerial vehicles (UAVs), integrated aerial platforms (IAPs) containing multiple UAVs do not suffer from underactuation and can move omnidirectionally in six dimensions, providing a basis for constructing aerial manipulation platforms. Compared to single UAVs, multi-UAV IAPs are also advantageous in terms of payload and fault-tolerance capacity, making them promising candidates as platforms with integrated-response, observation, and strike capabilities. Herein, an IAP structure design containing three sub-UAVs connected in a star-like configuration is presented. This form of integration enables the IAP, as a whole, to simultaneously adjust its position and attitude in six dimensions. The dynamics of the overall system of the IAP are modeled. On this basis, an overall system controller is designed. To simplify control, based on stability of cascaded system, the rotational motion of the sub-UAVs is treated as a inner-loop subsystem, whereas the overall motion of the IAP is seen as a outer-loop subsystem. Because the configuration space of the sub-UAVs is non-Euclidean, a controller is designed for the outer-loop subsystem based on model predictive control on the manifold. Subsequently, the stability of the closed-loop system is demonstrated. Fieldbus technology is employed to design a real-time, scalable communication architecture for multiple sub-UAVs, followed by the development of a principle prototype of the multi-UAV IAP that consists of hardware and software systems. The effectiveness of the IAP design and control method is validated through simulation and real-world prototype-based tests. In the simulation and real-world tests, the proposed methodology can make the IAP system converge to the desired configuration at the presence of large initial configuration error. The same test scenario cannot be finished by a baseline PID controller. The advantage of the proposed control scheme in dealing with state and input constraints is shown via such tests.

## 1. Introduction

### Motivation and Background

Unmanned aerial vehicles (UAVs) are expected to play a significant role in future applications [1,2,3,4]. Most conventional UAVs are only equipped with observation capabilities. In recent years, aerial manipulators with manipulation and response capabilities have garnered increasing attention [5,6,7,8,9]. These robots can expand the functions of aerial vehicles (AVs) and enhance the mobility and response range of conventional task-oriented robotic systems, showing promise as an important tool for future combat operations.

Most aerial manipulators rely on micro AV (MAV) platforms. To improve energy efficiency during flight, the available MAVs are generally underactuated, that is, they are actuated by a three-dimensional (3D) torque and a one-dimensional thrust. This underactuated design allows MAVs to be controllable and energy-efficient during flight. However, underactuated MAVs face challenges in tracking any six-dimensional (6D) trajectory in the special Euclidean group SE(3); they often require the attitude trajectories and position trajectories to satisfy certain dynamic constraints. Moreover, the low payload capacity precludes most MAVs from generating adequate forces and torques during contact interaction with the environment. In fact, these characteristics make MAVs more suitable as observation vehicles, rather than manipulation vehicles.

A system comprising a swarm of multiple small UAVs is characterized by flexible formations, intervehicle coordination, and the ability to adapt to different environments and perform complex missions. Consequently, UAV-swarm systems constitute a current topical area of research [10,11,12,13]. However, the payload capacity of the individual UAVs in a conventional UAV swarm cannot be combined due to a lack of physical interaction between them. Therefore, these UAV swarms are unsuitable for manipulation and response missions. Herein, based on the above analysis, we present an integrated design featuring a group of physically connected UAVs. This design allows for full realization of the advantages of a swarm of UAVs while enhancing the payload and manipulation capacity of UAVs through their physical interaction.

An integrated aerial platform (IAP) containing multiple UAVs possesses unique advantages [14,15]. As shown in Lee’s work, by combining three under-actuated UAV together in a certain way, the system is capable of fully actuated [16]. Lee et. al. proposed a prototype of IAP based on PID controller [14]. The similar work includes [17,18]. Unlike designing a UAV with a completely new structure, constructing an IAP involves combining available UAVs, which is easier to achieve. As a whole, such an IAP can obtain an omnidirectional driving force by combining the forces and torques provided by its constituent UAVs (referred to as sub-UAVs), that is, it has six independent force-screw inputs. This feature enables an IAP to move in all directions, that is, simultaneously and independently change its 6D position and attitude. As a manipulation platform, an IAP can somewhat compensate for the deficiencies of single UAVs and is expected to achieve higher manipulation flexibility and capacity, with the potential to interact compliantly and with six degrees of freedom relative to the environment.

However, the mutual configuration and force coupling between the sub-UAVs of an IAP present a challenge for the design of this complex system. The input boundedness and the state constraints need to be carefully considered. Moreover, the configuration space of UAVs is a non-Euclidean manifold. Due to the ability of an IAP to move omnidirectionally, its geometric control needs to be investigated in its non-Euclidean manifold configuration space to maximize its potential motion flexibility. Scalable, reliable, and low-latency communication among multiple strongly coupled UAVs is also required for cooperative control. Wireless communication is used in conventional UAV swarms. However, wireless-communication delays are detrimental to the cooperative control of the sub-UAVs of an IAP under strong coupling constraints.

To address the above problems, an IAP structure containing three sub-UAVs is designed in this study. Based on stability of cascaded system, the equation of motion of the overall system is subjected to decomposition and order reduction. The attitude motion of the sub-UAVs is treated as a outer loop subsystem, whereas the motion in the task space is seen as a inner-loop subsystem. The controller of the outer-loop subsystem constitutes the flight controller of the individual sub-UVAs. Because the configuration space of the IAP is non-Euclidean, and inorder to deal with state and input constraints, a geometric controller is designed for the slow-variable subsystem based on MPC. The overall stability of the closed-loop system is demonstrated based on Lyapunov stability theory. The IAP design and control method are validated through simulation and real-world flight tests. A fieldbus-based master–slave communication architecture, along with a complementary protocol, is developed to enhance the real-time performance of the communication between the sub-UAVs of the system and to facilitate the expansion of the quantity of sub-UAVs in the IAP.

Overall, the contribution of this paper can be summarized as follows.

- A control scheme is carefully designed such that it is singularity-free and can deal with the non-Euclidean configuration space, state and input constraints, hence is suitable for the IAP system. The control scheme is designed based on model predictive control and the dynamics of the IAP.
- The stability of the IAP under the control of the proposed MPC-based controller along with the existing flight controller of each individual sub-UAV is proved. This provides theoretical basis for the development of the IAP.
- By developing the hardware and the software system of the IAP, the proposed control scheme is successfully implemented in a prototype of IAP. To the best of the authors’ knowledge, this is the first time that the geometric model predictive control-based control scheme was successfully implemented in the real IAP prototype. The advantage of the proposed control scheme is shown through the comparison.

The remaining of the paper consists of four sections. The configuration and the dynamic modeling of the IAP is proposed in Section 2. The controller design and the convergence analysis are presented in Section 3. The simulation and real-world tests of the proposed control scheme are shown in Section 4.

## 2. Configuration and Dynamic Modeling of IAP

**Figure 1:**
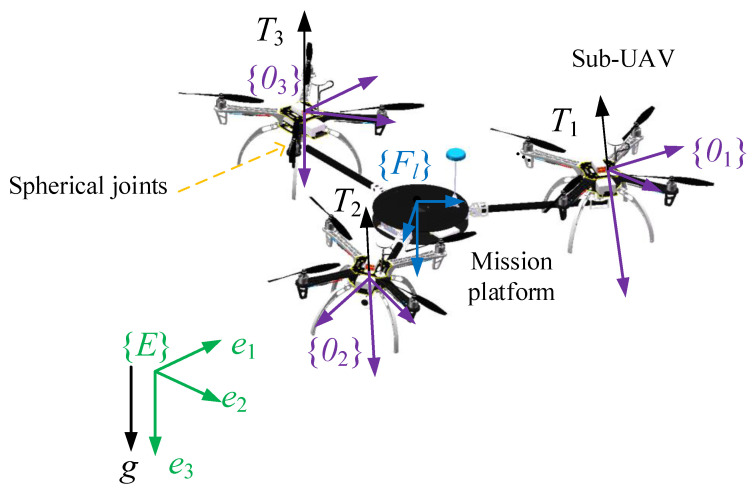
Schematic of an IAP with three sub-UAVs.

A dynamic model of the IAP is established using the following procedure. First, a world coordinate system $\left\{E\right\}$ fixed to the ground is constructed, followed by the establishment of a coordinate system $\left\{F_{1}\right\}$ fixed to the IAP at its overall center of mass. Then, a coordinate system $\left\{F_{2}\right\}$ fixed to the MP and with its origin located at the center of mass of the MP is constructed. Finally, for each sub-UAV, a body coordinate system $\left\{O_{i}\right\}$ (where i=1,2,⋯,n) fixed to it at its center of mass is established. Figure 1 is the schematic of an IAP with three sub-UAVs. In Figure 1, the three sub-UAVs connect the mission-platform via spherical joints. As have stated, the COM of each sub-UAV coincides with the center of spherical joints. Therefore, each sub-UAV can rotate around the spherical joints freely, and each sub-UAV is an under-actuated UAV, which means that it can generate a thrust and three torques. Because of this configuration, the thrust vector provided by each sub-UAV can be adjusted by adjusting the thrust magnitude and the attitude of each sub-UAV. By coordinately adjusting the thrust vector of each sub-UAV, the motion of the entire IAP can therefore be adjusted.

Thus, a dynamic model of the system can be derived from the Newton–Euler equation:

(1) $$M{\dot{V}}_{0}+CV_{0}+G=\left[\begin{array}{cc} R_{0} & 0\\ 0 & I \end{array}\right]u_{0}$$

where $V_{0}:=\left(v_{0},\omega_{0}\right)\in R{}^{6}$ (where $v_{0}\in R{}^{3}$ is the linear velocity in $\left\{E\right\}$ and $\omega_{0}\in R{}^{3}$ is the angular velocity in $\left\{F_{1}\right\}$ ) is the velocity of the overall system at its center of mass; $M\in R^{6\times 6}$, $C\in R^{6\times 6}$, and $G\in R^{6}$ are the mass, Coriolis, and gravitational acceleration matrices, respectively; $R_{0}\in \mathrm{SO}\left(3\right)$ is a rotation matrix of the MP; *I* is the identity matrix; and $u_{0}\in R^{6}$ is the resultant force and torque generated by the sub-UAVs described in $\left\{F_{1}\right\}$. The mass, Coriolis, and gravitational acceleration matrices are expressed as follows:

$$M=\left[\begin{array}{cc} m_{t} & 0\\ 0 & M_{t} \end{array}\right],C=\left[\begin{array}{cc} 0 & 0\\ 0 & -{\left(M_{t}\omega_{0}\right)}^{\wedge } \end{array}\right]$$

$$G=\left[\begin{array}{c} m_{t}ge_{3}\\ 0 \end{array}\right]$$

where

$$m_{t}=\sum_{i=0}^{n}m_{i}$$

$$M_{t}=M_{0}-\sum_{i=1}^{n}m_{i}{\hat{w}}_{i}\left({\hat{w}}_{i}-{\hat{w}}_{c}\right)$$

where $m_{i}$ denotes the mass of sub-UAV *i*, $m_{0}$ represents the MP’s mass, $M_{0}$ is the rotational inertia of the MP with respect to $\left\{F_{2}\right\}$, *g* is the gravitational acceleration, $w_{i}\in R^{3}$ is the position of sub-UAV *i* in $\left\{F_{2}\right\}$, $w_{c}\in R^{3}$ represents the coordinates of the center of mass of the whole system in $\left\{F_{2}\right\}$, and $e_{3}={\left(0,0,1\right)}^{T}$. Here, vector *a* is defined as follows: $a={\left(a_{1},a_{2},a_{3}\right)}^{T}\in R^{3}$. Let $\hat{a}$ be the corresponding antisymmetric matrix of *a*, as shown below:

$$\hat{a}=\left[\begin{array}{ccc} 0 & -a_{3} & a_{2}\\ a_{3} & 0 & -a_{1}\\ -a_{2} & a_{1} & 0 \end{array}\right]$$

A dynamic model of the system can be obtained using the equations below:

(2) $${\dot{p}}_{0}=v_{0},\;{\dot{R}}_{0}=R_{0}{\hat{\omega }}_{0}$$

where $p_{0}\in R{}^{3}$ is the position of the center of mass of the overall system in $\left\{E\right\}$.

Because the connecting spherical hinge is located at the center of mass of each sub-UAV in the IAP, the rotational motion of each sub-UAV follows the equations below:

(3) $${\dot{R}}_{i}=R_{i}{\dot{\omega }}_{i},\;{\dot{\omega }}_{i}=M_{i}^{-1}\left(\tau_{i}-{\hat{\omega }}_{i}M_{i}\omega_{i}\right)$$

where $R_{i}\in \mathrm{SO}\left(3\right)$ and $\omega_{i}\in R^{3}$ are the attitude rotation matrix and angular velocity of sub-UAV *i*, respectively, and $\tau_{i}\in R^{3}$ is the input torque of sub-UAV *i* (where *i* = 1, 2,..., *n*).

Here, $T_{i}\in R$ is used to denote the thrust generated by sub-UAV *i*. Based on the quadrotor structure, the thrust acting on the body coordinate system and points in the negative direction of $R_{i}e_{3}$. Therefore, the thrust vector generated by each sub-UAV can be expressed as:

(4) $$\gamma_{i}=R_{0}^{T}R_{i}e_{3}T_{i}$$

Based on the results presented above, the input $u_{0}$ in Equation (1) can be expressed as follows:

(5) $$u_{0}=\left[\begin{array}{ccc} I & I & \begin{array}{cc} \ldots & I \end{array}\\ {\hat{l}}_{1} & {\hat{l}}_{2} & \begin{array}{cc} \ldots & {\hat{l}}_{n} \end{array} \end{array}\right]\left[\begin{array}{c} \gamma_{1}\\ \gamma_{2}\\ \begin{array}{c} \ldots \\ \gamma_{n} \end{array} \end{array}\right]:=B\gamma$$

where $l_{i}$ is a distance defined by $w_{i}-w_{c}$, ( i=1,2,…,n).

Equations (1)–(4) collectively constitute the dynamic model of the IAP system.

For the IAP system, as the input of each sub-UAV is bounded, the 6D wrench acting on the entire IAP system is also bounded. We express such boundedness as,

(6) $$\begin{array}{cc} \; & u_{0}=\left(F_{0},\tau_{0}\right)\in U=\{\tau_{0}={\left(\tau_{0,x},\tau_{0,y},\tau_{0,z}\right)}^{T}\in R^{3}:∥\tau_{0}∥\leq \tau_{m}\}\\ \; & \;\times \{F_{0}={\left(F_{0,x},F_{0,y},F_{0,z}\right)}^{T}\in R^{3}:F_{0,x}^{2}+F_{0,y}^{2}\leq F_{m,h}^{2},|F_{0,z}+m_{t}g|\leq F_{m,z}\} \end{array}$$

## 3. Controller Design and Analysis

### 3.1. Overall Architecture

Based on the dynamic model, the attitude motion of each sub-UAV in the IAP is unaffected by the overall motion of the IAP. Therefore, the stability theorem of cascaded system is adopted to design a controller [19]. Specifically, the motion of the overall system is viewed as a inner-loop subsystem, whereas the attitude motion of each sub-UAV is treated as a outer-loop subsystem. As a result of this separation, the motion control of the overall system constitutes the outer loop of the attitude control of the sub-UAVs. Mature attitude motion control methods for UAVs are available and can be used directly. Here, focus is placed primarily on the motion control design of the outer loop of the IAP. The overall closed-loop system of the IAP can be proven to be stable under the action of a controller designed using this approach.

**Figure 2:**
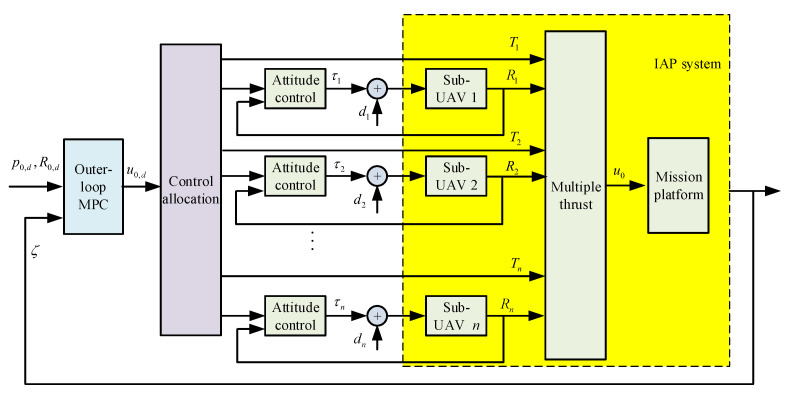
Architecture of the overall IAP controller based on geometric model predictive control.

The overall controller of the IAP is composed of model predictive control (MPC) on the manifold. MPC outputs a 6D force/torque $u_{0}$. The first three components of $u_{0}$ are force vectors, while its last three components are 3D torque vectors. Figure 2 shows the overall architecture of the controller. In the overall control architecture, the outerloop is a Model Predictive Control which outputs the 6D desired wrench $u_{0,d}\in R^{6}$. The 6D wrench is then transformed into the commanded attitude $R_{i}\in SO\left(3\right)$ and thrust $T_{i}\in R$ of each sub-UAV via the control allocation. By adopting such an architecture, the attitude controller of each sub-UAV is kept, while the outerloop MPC of the entire IAP can be used to deal with the state and input constraints of the IAP. The architecture supports the faster design and the implementation of the new controller while preserving the existing controllers.

In outer-loop control, the 6D wrench generated by position and attitude control needs to be converted to the thrust vectors of the sub-UAVs. The input and output of this control, distribution, and mapping process are the control quality $u_{0}\in R{}^{6}$ and the thrust vector of each sub-UAV, respectively. The following equation can be used to determine the thrust vector of each sub-UAV:

(7) $$\Lambda :=-B^{T}{\left[BB^{T}\right]}^{-1}u_{0}$$

where $\Lambda ={\left({\gamma_{1}}^{T},{\gamma_{2}}^{T},{\gamma_{3}}^{T}\right)}^{T}\in R{}^{9}$, and $B^{T}{\left[BB^{T}\right]}^{-1}\in R{}^{9\times 6}$ is the Moore–Penrose pseudoinverse of $B\left(d\right)$.

### 3.2. Outer Loop of the MPC Controller

The discrete time-series method is often employed to solve MPC problems. Given a sampling interval $\delta_{t}$, a sampling time series $t_{k}$ (where k∈N) can be defined. At sampling instant $t_{k}$, MPC is established via the solution of the optimal control problem as:

(8) $$\begin{array}{cc} \; & \min_{u_{0}\left(s\right)}J\left(\zeta ,u_{0}\right)=\;V_{r}\left(\zeta_{r}\left(t_{k}+\Gamma \right)\right)+V_{t}\left(\zeta_{t}\left(t_{k}+\Gamma \right)\right)+\\ \; & \int_{t_{k}}^{t_{k}+\Gamma }\left(N_{r}\left(\zeta_{r}\left(s\right),\tau_{0}\left(s\right)\right)+N_{t}\left(\zeta_{t}\left(s\right),F_{0}\left(s\right)\right)\right)ds\\ \; & s.t.\;\dot{\zeta }\left(s\right)=f\left(\zeta ,u_{0}\left(s\right)\right),\zeta \left(t_{k}\right)=\zeta \left(t_{k}\right),\\ \; & \zeta \in X\times V,u_{0}\left(s\right)\in U,\zeta \in \Omega_{r}\times \Omega_{t} \end{array}$$

where

$$\Gamma >\delta_{t}$$

is the predictive horizon of MPC; $\zeta_{r}=\left(R_{0,e},\omega_{0}\right)$ is the state of attitude motion; $\zeta_{t}=\left(p_{0,e},v_{0}\right)$ , is the state of displacement motion; $V_{r}\left(\zeta_{r}\right)$, $V_{t}\left(\zeta_{t}\right)$, $N_{r}\left(\zeta_{r},\tau_{0}\right)$, and $N_{t}\left(\zeta_{t},F_{0}\right)$ are all positive-definite functions (their definitions are described in detail later in Propositions 1 and 2), X×V are the admissible state set, and $\Omega_{r}$ and $\Omega_{t}$ are the sets of terminal constraints for the position and attitude channels, respectively, which are used to ensure the stability of the overall IAP system under MPC and the solvability of MPC (they are similarly described in detail later in Propositions 1 and 2).

#### 3.2.1. Terminal Set Constraints and Terminal Control of Attitude Motion

For the attitude control of the slow-time varying system, the angular velocity command is defined as follows:

(9) $$\omega_{0,d}=-k_{1}e_{R,0}$$

where $k_{1}$ is a positive constant, and $e_{R,0}=\frac{1}{2}{\left(R_{0,e}-R_{0,e}^{T}\right)}^{\vee }$.

Based on Equation (9), a torque control law $\tau_{0}$ is designed as follows:

(10) $$\tau_{0}=-k_{2}M_{t}\omega_{0,e}+\omega_{0}\times M_{t}\omega_{0}+M_{t}{\dot{\omega }}_{0,d}$$

where $k_{2}$ is a positive constant, and $\omega_{0,e}=\left(\omega_{0}-\omega_{0,d}\right)$.

Substituting Equation (9) into the equation of motion gives the following:

(11) $${\dot{\omega }}_{0,e}=-k_{2}\omega_{0,e}$$

The attitude state of the MP can be expressed using $\zeta_{r}={\left(e_{R,0}^{T},\omega_{0}^{T}\right)}^{T}$ and $\xi_{r}={\left(e_{R,0}^{T},\omega_{0,e}^{T}\right)}^{T}$, and can be reorganized into the following equation:

(12) $$\xi_{r}=\left[\begin{array}{cc} I & 0\\ k_{1}I & I \end{array}\right]\zeta_{r}:=A_{r}\zeta_{r}$$

Then, the following proposition can be defined to analyze the feasibility and convergence of attitude control.

**Proposition** **1.** *The equation of attitude motion of the MP is analyzed. A set of terminal constraints in MPC is defined as follows: $\Omega_{r}=\{\zeta_{r}:V_{r}\left(\zeta_{t}\right)=\frac{1}{2}tr\left(I-R_{0,e}\right)+\frac{1}{2}h_{11}∥\omega_{0}+k_{1}e_{R,0}{∥}^{2}\leq \epsilon_{r}:=\frac{\tau_{m}^{2}}{\max\left(\frac{L_{2}}{2},\frac{2L_{1}}{h_{11}}\right)}\}$, where $h_{11}$ is a positive constant. If $\zeta_{r}\left(t_{k}+\Gamma \right)\in \Omega_{r}$, a torque control law $\tau_{0}\left(s\right),s\in \left(t_{k}+\Gamma ,t_{k+1}+\Gamma \right)]$ can be derived from (10). Selecting suitable control parameters allows the following conclusions to be reached for all $s\in (t_{k}+\Gamma ,t_{k+1}+\Gamma )]$,*

*(1)*  *$\Omega_{r}$ is invariant,*
*(2)*  *${\dot{V}}_{r}+N_{r}\left(\xi_{r},\tau_{0}\right)\leq 0$, where*
$$\begin{array}{c} N_{r}=\zeta_{r}^{T}A_{r}^{T}\left[\begin{array}{cc} q_{11}I & 0\\ 0 & q_{12}I \end{array}\right]A_{r}\zeta_{r}+\tau_{0}^{T}r_{1}\tau_{0}\\ :=\zeta_{t}^{T}Q_{r}\zeta_{t}+\tau_{0}^{T}R_{r}\tau_{0} \end{array}$$
*where $q_{11}$, $q_{12}$, and r are all positive constants.*
*(3)*  *$\tau_{0}\in S_{6}$ holds for all $\zeta_{r}\in \Omega_{r}$.*

The proof can be obtained by observing $V_{r}$ and the evolution of ${\dot{V}}_{r}$. This process is omitted here.

#### 3.2.2. Terminal Set Constraints and Terminal Control of Position Motion

For the position control of the MP, the velocity command is first defined as follows:

(13) $$v_{0,d}=-k_{3}p_{0,e}$$

where $k_{3}$ is a positive constant.

Based on Equation (13), a feedback control law for force vectors is further designed, as shown below:

(14) $$F_{0}=-k_{4}m_{t}R_{0}^{T}v_{0,e}-R_{0}^{T}m_{t}ge_{3}+R_{0}^{T}m_{t}{\dot{v}}_{0,d}$$

where $k_{4}$ is a positive constant, and $v_{0,e}=v_{0}-v_{0,d}$.

The position state of the MP can be expressed using $\zeta_{t}={\left(p_{0,e}^{T},v_{0}^{T}\right)}^{T}$, and $\xi_{t}=(p_{0,e}^{T}$$v_{0,e}^{T}{)}^{T}$, which can be reorganized into the following equation:

(15) $$\xi_{t}=\left[\begin{array}{cc} I & 0\\ k_{3}I & I \end{array}\right]\zeta_{t}:=A_{t}\zeta_{t}$$

The following proposition is defined to analyze the convergence of position control.

**Proposition** **2.** *The equation of position motion of the MP is analyzed. The attitude of the MP is subject to $e_{3}^{T}R_{0}e_{3}\geq L_{l}$, where $L_{l}$ is a positive constant that is less than 1. A set of terminal constraints in MPC is defined as follows:*

$$\Omega_{t}=\left\{\zeta_{t}:V_{t}\left(\zeta_{t}\right):=\frac{1}{2}\zeta_{t}^{T}H_{t}\zeta_{t}\leq_{t}^{2}\right\}$$

*where $H_{t}$ is a positive-definite matrix,*

$$H_{t}=A_{t}^{T}\left[\begin{array}{cc} h_{21}I & 0\\ 0 & h_{22}I \end{array}\right]A_{t}$$

*where $h_{21}$ and $h_{22}$ are positive constants, and*

$$\epsilon_{t}=\min\left(\frac{F_{m,z}-\Delta_{F}}{\sqrt{2}m_{t}\left(\frac{k_{3}+k_{4}}{\sqrt{h_{21}}}+\frac{k_{3}^{2}}{\sqrt{h_{22}}}\right)},\frac{F_{m,h}-m_{t}g\sqrt{1-L_{l}^{2}}}{\sqrt{2}m_{t}\left(\frac{k_{3}+k_{4}}{\sqrt{h_{11}}}+\frac{k_{3}^{2}}{\sqrt{h_{22}}}\right)}\right)$$

*When $\zeta_{t}\left(t_{k}+\Gamma \right)\in \Omega_{t}$t, Equations (14) can be used to generate a control law for force vectors, $F_{0}\left(s\right),s\in \left(t_{k}+\Gamma ,t_{k+1}+\Gamma \right)]$. Selecting suitable controller parameters allows the following propositions to hold for all $s\in (t_{k}+\Gamma ,t_{k+1}+\Gamma )]$,*

*(1)*  *$\Omega_{t}$ is invariant.*
*(2)*  *${\dot{V}}_{t}+N_{t}\leq 0$, where*
$$\begin{array}{c} N_{t}=\zeta_{t}^{T}A_{t}^{T}\left[\begin{array}{cc} q_{21}I & 0\\ 0 & q_{22}I \end{array}\right]A_{t}\zeta_{t}+\\ {\left(F_{0}+m_{t}R_{0}^{T}ge_{3}\right)}^{T}r_{21}\left(F_{0}+m_{t}R_{0}^{T}ge_{3}\right) \end{array}$$
*where $q_{21}$, $q_{22}$, and $r_{21}$ are all positive constants.*
*(3)*  *$F_{0}\in S_{5}$ holds for all $\zeta_{t}\in \Omega_{t}$.*

The proof process is omitted here.

### 3.3. Solvability and Stability of the Closed Loop IAP

**Theorem** **1.** *Consider the IAP outer-loop sub-system (1). The controller is the MPC solved from the finite time optimal problem (8). Suppose (8) is solvable at initial time. Then the closed loop outer-loop system is asymptotically stable.*

**Proof.**  The solvability of the MPC can be proved recursively. Assume the optimal control problem is solvability at instant $t_{k}$, and the sulution is expressed as $u_{0}^{*}\left(s\right),s\in \left[t_{k},t_{k}+\Gamma \right]$. Then According to definition of the constraints, at next sampling instant $t_{k}$, the state ζ is always in the terminal region under $u_{0}^{*}\left(t_{k}\right)$.

In the time interval $\left[t_{k+1},t_{k+1}+\Gamma \right]$, the following solution for (8) can be constructed,

(16) $$u_{0,f}=\left\{\begin{array}{c} u_{0}^{*}\left(s\right),s\in \left[t_{k+1},t_{k}+\Gamma \right]\\ u_{0,ter}\left(s\right),s\in \left(t_{k}+\Gamma ,t_{k+1}+\Gamma \right] \end{array}\right.$$

where the control $u_{0,ter}=\left(F_{0,ter},\tau_{0,ter}\right)$ is given by terminal control law (10) and (14). From Propositions 1 and 2, it is seen that under the control of $u_{0,ter}$ defined by (10) and (14), $u_{0,ter}\left(s\right)\in U$ for all $s\in (t_{k}+\Gamma ,t_{k+1}+\Gamma ]$, and state will be kept in terminal region at time $t_{k+1}+\Gamma$. Therefore, under the control $u_{0,ter}$ we have,

(17) $$\zeta \left(t_{k+1}+\Gamma \right)\in \Omega_{r}\times \Omega_{t}$$

In summary, we say that $u_{0,f}\left(s\right),s\in \left[t_{k+1},t_{k+1}+\Gamma \right]$ is a feasible solution of (8). The solvability of (8) is therefore provided recursively.

The second part is the convergence proof of the system under MPC. For this purpose, define the following Lyapunov candidate,

(18) $$V=J(\zeta ,u_{0})$$

We can derive the difference of *V* from $t_{k}$ to $t_{k+1}$ as,

(19) $$\begin{array}{cc} \; & \Delta V=V\left(t_{k+1}\right)-V\left(t_{k}\right)\\ \; & =\int_{t_{k+1}}^{t_{k+1}+\Gamma }\left[N_{r}\left(\zeta_{r}\left(s\right),\tau_{0}\left(s\right)\right)+N_{t}\left(\zeta_{t}\left(s\right),F_{0}\left(s\right)\right)\right]ds\\ \; & -\int_{t_{k}}^{t_{k}+\Gamma }\left[N_{r}\left(\zeta_{r}\left(s\right),\tau_{0}\left(s\right)\right)+N_{t}\left(\zeta_{t}\left(s\right),F_{0}\left(s\right)\right)\right]ds\\ \; & +V_{r}\left(\zeta_{r}\left(t_{k+1}+\Gamma \right)\right)+V_{t}\left(\zeta_{t}\left(t_{k+1}+\Gamma \right)\right)-\\ \; & V_{r}\left(\zeta_{r}\left(t_{k}+\Gamma \right)\right)-V_{t}\left(\zeta_{t}\left(t_{k}+\Gamma \right)\right)\\ \; & =\int_{t_{k}+\Gamma }^{t_{k+1}+\Gamma }\left[N_{r}\left(\zeta_{r}\left(s\right),\tau_{0}\left(s\right)\right)+N_{t}\left(\zeta_{t}\left(s\right),F_{0}\left(s\right)\right)\right]ds\\ \; & -\int_{t_{k}}^{t_{k+1}}\left[N_{r}\left(\zeta_{r}\left(s\right),\tau_{0}\left(s\right)\right)+N_{t}\left(\zeta_{t}\left(s\right),F_{0}\left(s\right)\right)\right]ds\\ \; & +V_{r}\left(\zeta_{r}\left(t_{k+1}+\Gamma \right)\right)+V_{t}\left(\zeta_{t}\left(t_{k+1}+\Gamma \right)\right)-\\ \; & V_{r}\left(\zeta_{r}\left(t_{k}+\Gamma \right)\right)-V_{t}\left(\zeta_{r}\left(t_{k}+\Gamma \right)\right) \end{array}$$

We integrate ${\dot{V}}_{r}+{\dot{V}}_{t}+N_{t}+N_{r}$ in the time interval $\left[t_{k}+\Gamma ,t_{k+1}+\Gamma \right]$. Considering Proposition 1 and 2 we have,

(20) $$\begin{array}{cc} \; & \int_{t_{k}+\Gamma }^{t_{k+1}+\Gamma }\left[N_{r}\left(\zeta_{r}\left(s\right)\right)+N_{t}\left(\zeta_{t}\left(s\right)\right)\right]ds\\ \; & +V_{r}\left(\zeta_{r}\left(t_{k+1}+\Gamma \right)\right)+V_{t}\left(\zeta_{t}\left(t_{k+1}+\Gamma \right)\right)-\\ \; & V_{r}\left(\zeta_{r}\left(t_{k}+\Gamma \right)\right)-V_{t}\left(\zeta_{t}\left(t_{k}+\Gamma \right)\right)\leq 0 \end{array}$$

Substituting (20) into (19) yields,

(21) ΔV≤0

Then we can conclude that the closed outer-loop subsystem is asymptotically stable. □

**Remark** **1.** *Theorem 1 only shows the stability of the closed outer loop subsystem. It shows that the geometric model predictive control for the outer loop subsystem is always solvable if it is solvable at initial time. Actually, Theorem 1 reflects the asymptotical stability of the system without considering the attitude tracking error of each sub-UAV, i.e., assuming the tracking error of each sub-UAV is zero. One can imagine that in this case, the equivalent input to the outer loop subsystem is exactly the same with the resultant force and torque generated by the sub-UAVs. This is an ideal condition as the attitude tracking error of each sub-UAV always exists in actual systems. However, for the real system we can conclude that the entire system is stable if the attitude tracking controller of each sub-UAVs is stable.*

Then we consider the attitude tracking controller of each sub-UAV. The control vector $u_{0}$ is obtained by solving the finite-time optimal control problem at each time, as shown in Equation (8). Here, the attitude control of each sub-UAV is assumed to be exponentially stable. This is easy to realize as there has been mature control technologies for the attitude control of sub-UAVs [20,21]. Then, based on Theorem 1, in conjunction with stability of the system in cascade [19,20], the overall closed-loop system is asymptotically stable, and MPC is recursively solvable at each time.

**Remark** **2.** *It is noted that the cost function in (8) does not reply on any local coordinate of the attitude. Therefore, the controller of the outer-loop system is singularity-free. Moreover, if the inner-loop subsystem which reflects the rotational motion of the system is adequate, then the closed-loop entire system is also singularity free.*

## 4. Simulation and Real-World Tests

### 4.1. Simulation System Construction

Prior to real-world flight tests, the closed-loop control of the IAP was first simulated using the Robot Operating System (ROS) in an Ubuntu 18.04 environment. An ROS node was written based on (1) and (3) to simulate the body of the IAP. Perturbations were added to the simulated model of the body of the IAP to mimic uncertain disturbances in real-world flight. The input of the body simulation node consisted of the thrust vectors generated by the three sub-UAVs, and its output comprised the position, linear and angular velocities, and attitude of the MP of the IAP. In the simulation test, the remaining ROS nodes were used as interface nodes to receive commands from a remote control (RC) and transform them into the expected position and attitude of the multi-UAV IAP.

### 4.2. Simulation Results

**Figure 3:**
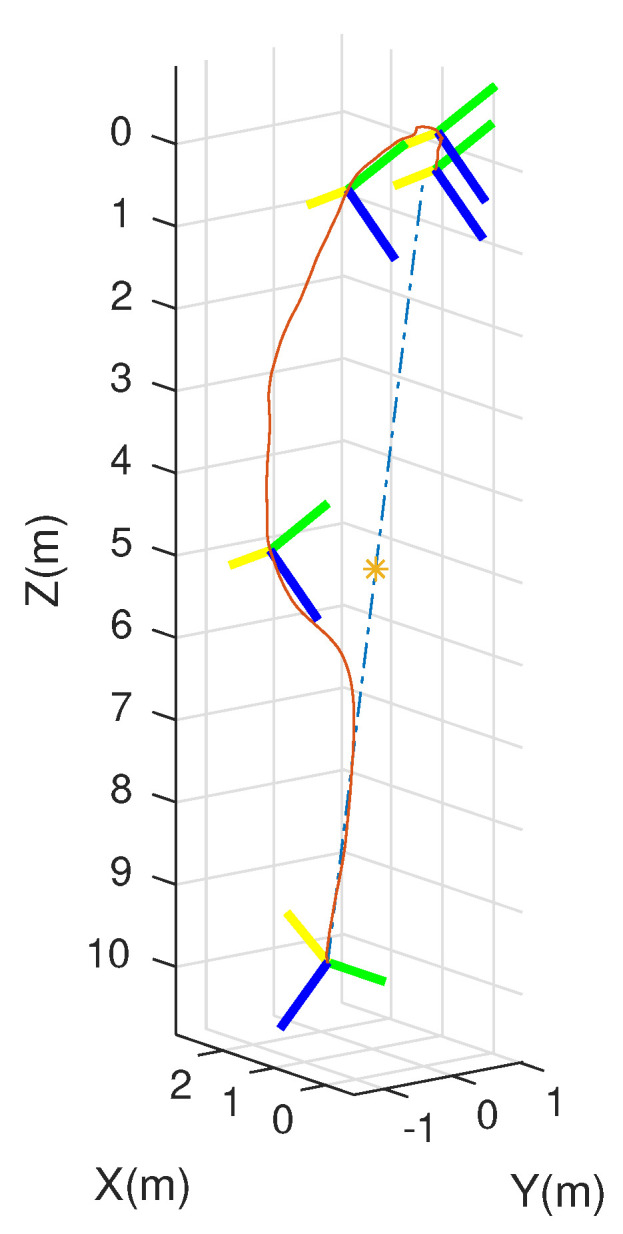
The configuration path of the mission-platform. (the x-, y-, and z-axes are shown in yellow, green, and blue, respectively).

**Figure 4:**
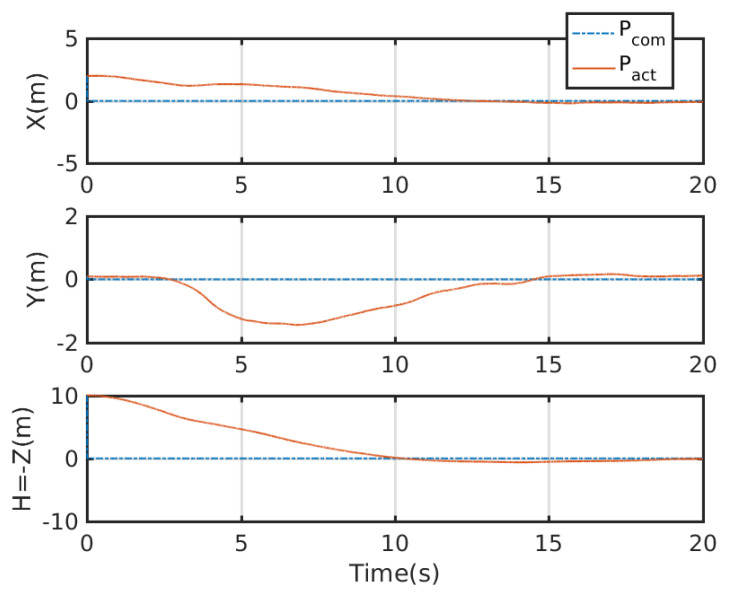
Overall position evolution of the IAP.

**Figure 5:**
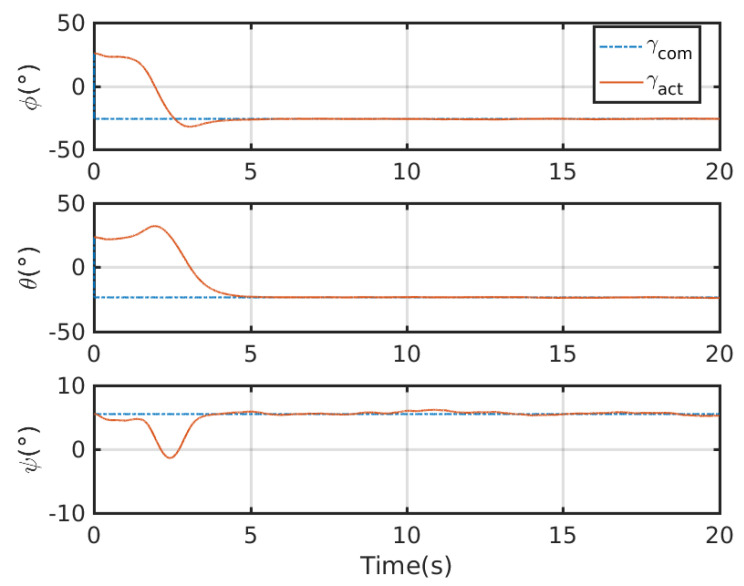
Overall attitude evolution of the IAP.

**Figure 6:**
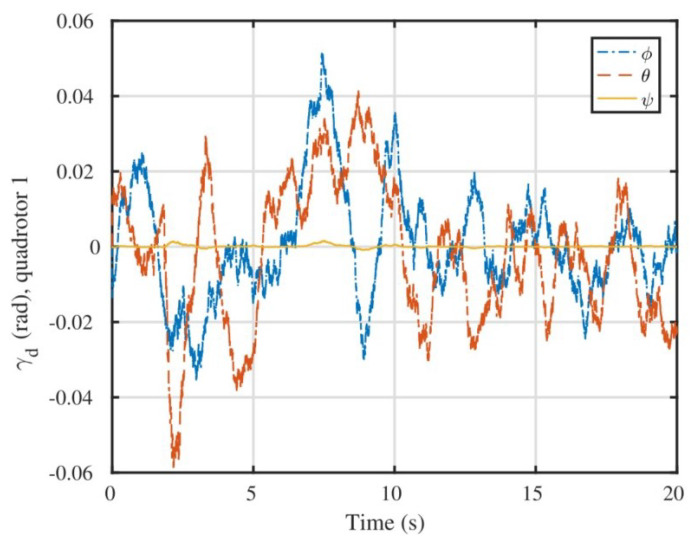
Commanded attitude of sub-UAV 1 expressed in Euler angles.

An integrated platform with six sub-UAVs was simulated under the MPC described earlier in the simulation test. The initial and expected positions of the simulated IAP were set to (2, 0.05, 10) and (0, 0, 0) m, respectively, and its initial and expected attitudes were set to $R_{0}=\exp\left(0.6\left(\frac{1}{\sqrt{2}},\frac{1}{\sqrt{2}},0\right)\right)$ and $R_{0,d}=\exp\left(-0.6\left(\frac{1}{\sqrt{2}},\frac{1}{\sqrt{2}},0\right)\right)$, respectively. An obstacle (radius 1 m ) was considered at (1, 0.05, 5) m in the simulation test. Figure 3, Figure 4, Figure 5 and Figure 6 show the tracked position and attitude of the IAP. It is evident that under the geometric MPC described earlier, the IAP reached the expected position and attitude from its initial position and attitude in a stable manner by simultaneously adjusting the attitude and thrust commands for its sub-UAVs. Because the state and input constraints could be satisfied in MPC, the IAP was able to maintain a distance from the obstacle throughout the process, as shown in Figure 3. The position evolution of the IAP is shown in Figure 4. It also shows the position evolves from the initial position to the designed position while keeping distance from the obstacles. The attitude evolution is shown in Figure 5 which indicates the convergence of the attitude from initial value to desired value. As an example, the attitude evolution of sub-UAV 1 is shown in Figure 6. The attitude of the sub-UAV is adjusted according to the output of the outer-loop controller. Due to the varying of the attitude of sub-UAV 1, the thrust vector provided by each sub-UAV is also varying. In summary, the feasibility, correctness, and completeness of the written controller node and overall software architecture were validated using the simulation model, providing a basis for conducting real-world flight tests.

The feasibility, correctness, and completeness of the written controller node and overall software architecture were validated using the simulation model, providing a basis for conducting real-world flight tests.

### 4.3. Development of an IAP Prototype Consisting of Software and Hardware Systems

Based on the results presented above, a principle prototype of the IAP, consisting of software and hardware systems, was developed. The MP of the IAP was integrated with appropriate avionic devices, including a flight control board (FCB) , an on-board computer (OBC) , a RC receiver (RCR) , a Global Positioning System (GPS) receiver , an inertial measurement unit (IMU) sensor , and a data transmission radio (DTR) . The FCB needed to be installed as close to the center of mass of the MP as possible; it was used to receive the commands from the RC and the position and attitude data produced by the sensor fusion algorithm and to transmit the commands, along with position and attitude data, to the OBC through a Universal Serial Bus (USB) serial port. The DTR was primarily used to monitor and record flight status and communicate with the ground station. The main structural components of the IAP prototype were made of a carbon-fiber material. The range of motion of the spherical hinges in the prototype of IAP was $55^{\circ }$.

Commercially available small quadrotors were used as sub-UAVs in the prototype. Each sub-UAV was equipped with a complete set of avionic devices (e.g., an FCB, a GPS device, an RCR, and an IMU sensor). Therefore, the sub-UAVs could fly in an integrated manner when combined together and individually when separated from each other. The commercially available PX4 open-source autopilot was used to control the flight of the sub-UAVs.

**Figure 7:**
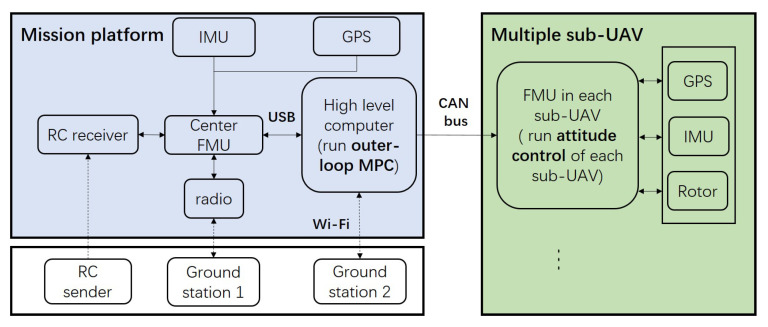
Hardware architecture of the assembly prototype.

To ensure the required reliable and real-time communication between the sub-UAVs during stable movement of the IAP, a system was developed based on the controller area network (CAN) bus architecture to enable mutual communication between the MP and the sub-UAVs. The OBC on the MP and the flight controller of each sub-UAV are considered nodes mounted on the CAN bus. This mode of communication can ensure reliable and real-time communication and facilitate the expansion of the number of sub-UAVs in the IAP. Figure 7 shows the overall hardware architecture of the IAP.

The ROS was used to design a software architecture for the IAP in an Ubuntu 18.04 environment. As a ROS node, the controller ran completely on the OBC installed on the MP. A one-to-many data communication protocol, termed Swarmlink, was also developed for the communication of the IAP. Corresponding encoding and decoding ROS nodes were set on both the MP and sub-UAV ends. Table 1 summarizes the relevant physical parameters of the developed overall IAP prototype and its sub-UAVs.

### 4.4. Real-World Test Results

The position and attitude of the IAP prototype with three sub-UAVs were tracked during tests. The initial and expected position in the real-world test were set to (0, 0, 0) and (−10, −8, −2) m, respectively The initial and expected attitudes in the real-world test were set to $R_{0}=I$ and R0,d=exp(−0.5(12,12,0)^), respectively. It is noted that there is noticeable initial error in the test. A typical PID controller cannot force the system stable at the presence of such initial error. The admissible input set in the real-world IAP is set as,

$$\begin{array}{cc} & U=\{\tau_{0}={\left(\tau_{0,x},\tau_{0,y},\tau_{0,z}\right)}^{T}\in R^{3}:∥\tau_{0}∥\leq 6Nm\}\\ & \;\times \{F_{0}={\left(F_{0,x},F_{0,y},F_{0,z}\right)}^{T}\in R^{3}:F_{0,x}^{2}+F_{0,y}^{2}\leq {\left(20N\right)}^{2},|F_{0,z}+m_{t}g|\leq 20\;N\} \end{array}$$

While the velocity constraint is also configured by the MPC-based scheme as $∥v_{0}∥\leq 2\;m/s$.

**Figure 8:**
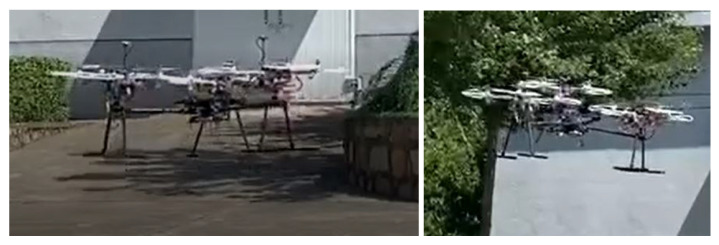
The IAP prototype maintaining a hover while changing its roll and pitch angles.

Figure 8 shows images captured during a flight test (the IAP maintains a stable hover at roll and pitch angles of approximately $0^{\circ }$ in the left image and at an inclined attitude in the right image). It is visually evident in Figure 8 that the IAP was able to maintain a hover while changing its roll and pitch angles. Such a maneuver cannot be achieved by conventional underactuated UAVs.

**Figure 9:**
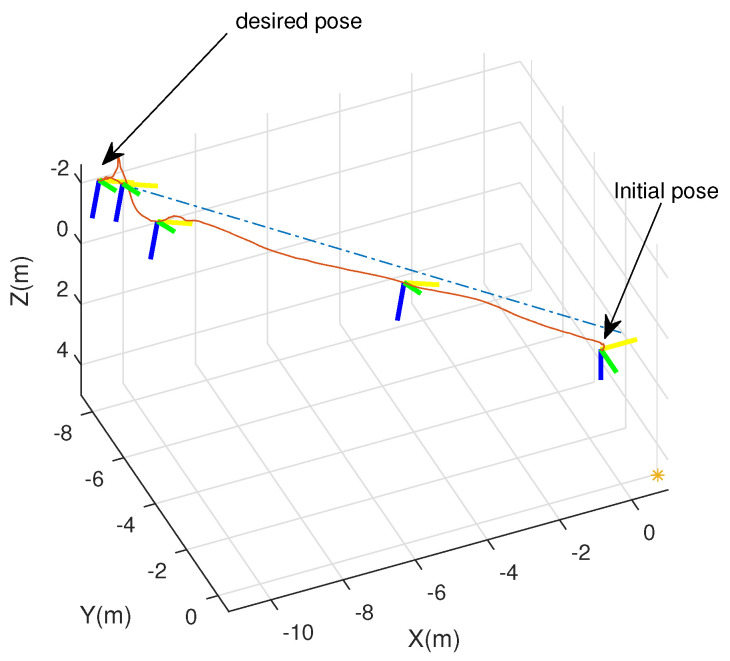
Position and attitude profiles tracked during real-world flight test. Note the big initial error here. A typical PID is difficult to stabilize the IAP in such condition as the controller may output large action which violates the input boundedness. In contrast, our proposed control scheme successfully stabilizes the system as it can deal with the state and input constraints.

**Figure 10:**
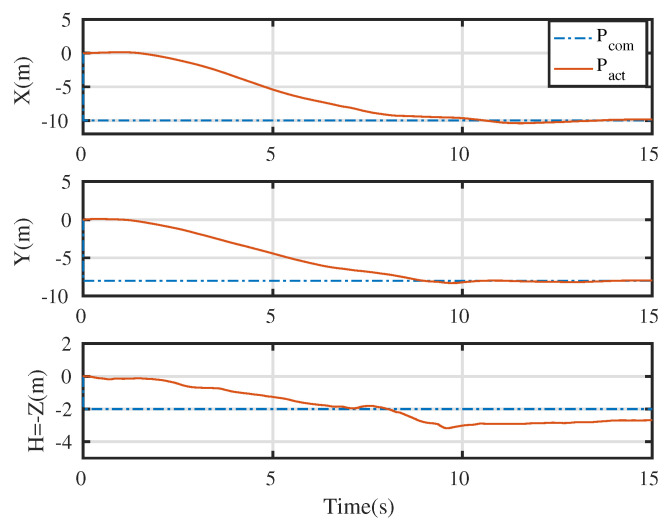
Position profile tracked during real-world flight test.

**Figure 11:**
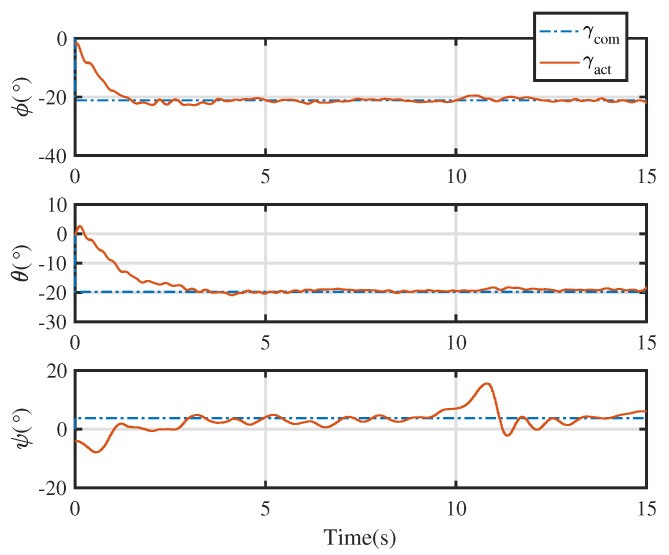
Attitude profiles tracked during real-world flight test.

Figure 9 show the tracking test results for the IAP prototype under the control of the proposed control scheme. The commanded attitude trajectory of the IAP was independent of its commanded position trajectory. Figure 10 shows the position trajectory of the IAP under the proposed control. Figure 11 shows the attitude trajectory of the IAP under the proposed control. For ease of display, Euler angles are used in the figure to depict the attitude. From Figure 9, Figure 10 and Figure 11 it is seen that the IAP converges to the desired configuration under the proposed control. It is evident that the roll, pitch, and yaw errors were all finally within reasonable range. The prototype was tested in an outdoor real-time kinematic GPS environment. As shown in Figure 10, the maximum horizontal and vertical position tracking errors were around 0.6 m, indicating that ideal tracking results were achieved for both attitude and position. It should be noted that there is large initial error in this test. Because the proposed control scheme can deal with input boundedness, the IAP remains stable during the entire flight.

The test results show that the IAP prototype was capable of moving omnidirectionally in six dimensions while tracking the position and attitude trajectories in a stable manner, without the need for the position and attitude trajectories to satisfy dynamic constraints. These capabilities are lacking in conventional underactuated UAVs. The real-world test results for the prototype also validate the stability of the designed controller.

**Remark** **3.** *We also test the IAP with the same scenario under a PID controller. However, as the PID controller could not deal with the state and input boundedness, the IAP prototype could not remain stable. Given the initial error, a PID controller may generate saturated action which may make the real-world IAP prototype unstable soon. The advantage of the proposed controller for the IAP in dealing with the input boundedness is thus verified through this experiment.*

## 5. Conclusions

This study presents an IAP configuration design containing three sub-UAVs, investigates the geometric control of the integrated multi-UAV system, and develops an IAP prototype that consists of software and hardware systems, providing a design and control basis for developing new multi-UAV IAPs. The IAP containing three UAVs connected through spherical hinges can move omnidirectionally in six dimensions and is an ideal choice for an aerial manipulation platform. This configuration can be altered to construct other IAPs. The designed geometric MPC controller can be effectively used to control the motion of the IAP. Its stability is validated both theoretically and experimentally. Our proposed control approach is easy for implementation and is globally effective. Compared with other control scheme, the proposed control scheme is capable of dealing with input and state constraints. Fieldbus technology is employed to construct a low-latency, high-reliability mode of communication for the IAP prototype. Subsequent real-world flight tests demonstrate the correctness and completeness of the developed software and hardware systems of the IAP prototype. This is the first time that the proposed control scheme was implemented in a real-world IAP. In future works, the motion planning of the proposed IAP for specific tasks will be considered. Development of IAP with different configurations is also with our interests.

## Figures and Tables

**Table 1:** Relevant physical parameters of the sub-UAVs and IAP prototype.

	Mass (kg)	Payload Capacity (kg)	Computing Unit
Sub-UAV	1.58	1.5	PX4 open-source FCB
IAP	6.24	3.02	Nvidia nano OBC and PX4 open-source FCB

## Data Availability

Not applicable.

## References

[1] Kim S.J., Lee D.Y., Jung G.P., Cho K.J. (2018). An origami-inspired, self-locking robotic arm that can be folded flat. Sci. Robot..

[2] Estrada M.A., Mintchev S., Christensen D.L., Cutkosky M.R., Floreano D. (2018). Forceful manipulation with micro air vehicles. Sci. Robot..

[3] Xilun D., Pin G., Kun X., Yushu Y. (2019). A review of aerial manipulation of small-scale rotorcraft unmanned robotic systems. Chin. J. Aeronaut..

[4] Ruggiero F., Lippiello V., Ollero A. (2018). Aerial manipulation: A literature review. IEEE Robot. Autom. Lett..

[5] Jimenez-Cano A.E., Sanchez-Cuevas P.J., Grau P., Ollero A., Heredia G. (2019). Contact-based bridge inspection multirotors: Design, modeling, and control considering the ceiling effect. IEEE Robot. Autom. Lett..

[6] Orsag M., Korpela C., Bogdan S., Oh P. (2017). Dexterous aerial robots—Mobile manipulation using unmanned aerial systems. IEEE Trans. Robot..

[7] Zhang G., He Y., Dai B., Gu F., Yang L., Han J., Liu G., Qi J. (2018). Grasp a moving target from the air: System & control of an aerial manipulator. Proceedings of the 2018 IEEE International Conference on Robotics and Automation (ICRA).

[8] Lippiello V., Fontanelli G.A., Ruggiero F. (2018). Image-based visual-impedance control of a dual-arm aerial manipulator. IEEE Robot. Autom. Lett..

[9] Yu Y., Wang K., Guo R., Lippiello V., Yi X. (2021). A framework to design interaction control of aerial slung load systems: Transfer from existing flight control of under-actuated aerial vehicles. Int. J. Syst. Sci..

[10] Welde J., Paulos J., Kumar V. (2021). Dynamically feasible task space planning for underactuated aerial manipulators. IEEE Robot. Autom. Lett..

[11] Yu Y., Li P., Gong P. (2020). Finite-time geometric control for underactuated aerial manipulators with unknown disturbances. Int. J. Robust Nonlinear Control..

[12] Park S., Lee J., Ahn J., Kim M., Her J., Yang G.H., Lee D. (2018). ODAR: Aerial Manipulation Platform Enabling Omnidirectional Wrench Generation. IEEE/ASME Trans. Mechatron..

[13] Park S., Lee Y., Heo J., Lee D. Pose and posture estimation of aerial skeleton systems for outdoor flying. Proceedings of the 2019 International Conference on Robotics and Automation (ICRA).

[14] Nguyen H., Park S., Park J., Lee D. (2018). A Novel Robotic Platform for Aerial Manipulation Using Quadrotors as Rotating Thrust Generators. IEEE Trans. Robot..

[15] Yu Y., Shi C., Shan D., Lippiello V., Yang Y. (2022). A Hierarchical Control Scheme for Multiple Aerial Vehicle Transportation Systems with Uncertainties and State/Input Constraints. Appl. Math. Model..

[16] Lee T. (2018). Geometric Control of Quadrotor UAVs Transporting a Cable-Suspended Rigid Body. IEEE Trans. Control Syst. Technol..

[17] Six D., Briot S., Chriette A., Martinet P. (2018). The Kinematics, Dynamics and Control of a Flying Parallel Robot with Three Quadrotors. IEEE Robot. Autom. Lett..

[18] Sanalitro D., Tognon M., Cano A.J., Cortes J., Franchi A. (2022). Indirect Force Control of a Cable-Suspended Aerial Multi-Robot Manipulator. IEEE Robot. Autom. Lett..

[19] Kendoul F. (2009). Nonlinear Hierarchical Flight Controller for Unmanned Rotorcraft: Design, Stability, and Experiments. J. Guigance Control Dyn..

[20] Yu Y., Ding X. (2016). A Global Tracking Controller for Underactuated Aerial Vehicles: Design, Analysis, and Experimental Tests on Quadrotor. IEEE/ASME Trans. Mechatron..

[21] Lee T. Geometric Tracking Control of the Attitude Dynamics of a Rigid Body on SO(3). Proceedings of the American Control Conference.

